# An examination of the relationship between satisfaction with overactive bladder (OAB) treatment and the doctor–patient gender: A questionnaire‐based single‐institution study

**DOI:** 10.1002/bco2.236

**Published:** 2023-03-25

**Authors:** Yukiko Kouchi, Akihiko Ozaki, Yudai Kaneda, Divya Bhandari, Kazuma Saito, Hiroaki Shimmura

**Affiliations:** ^1^ Department of Urology Jyoban Hospital of Tokiwa Foundation Iwaki Japan; ^2^ Department of Breast and Thyroid Surgery Jyoban Hospital of Tokiwa Foundation Iwaki Japan; ^3^ School of Medicine Hokkaido University Sapporo Japan; ^4^ School of Political Science and Economics Waseda University Tokyo Japan

**Keywords:** OABSS, overactive bladder, satisfaction, urologists

## Abstract

Overactive bladder (OAB) significantly reduces quality of life. The primary goal of this study was to determine whether the gender combination of patient and physician may be associated with satisfaction with OAB treatment. This questionnaire survey was conducted at Jyoban Hospital. We considered the adult patients aged 18 years or older who attended the outpatient office of the urology department of the hospital, were diagnosed with OAB and had been taking anticholinergics or β3‐receptor stimulants, or both, for at least 3 months. In addition to the OAB treatment satisfaction, the questionnaire covered OABSS, IPSS, oral medications, effectiveness of OAB treatment, response to OAB symptoms, and the medium and extent of information collection. A total of 147 patients participated in the study. In summary, 91 (61.9%) were male, and the mean age was 73.5 years. Compared to when the gender of doctor and patient was not the same, female patients tended to be significantly more satisfied when they were treated by female doctors (OR 10.79, 95% CI 1.27–92.05). On the other hand, no similar trend was observed when male patients were treated by male doctors (OR 1.26, 95% CI 0.25–6.34). In the present study, which examined doctor–patient gender combinations in satisfaction with OAB treatment, as hypothesized, satisfaction was higher for female doctor–female patient combinations compared to different doctor–patient genders. A notable fact was that similar associations were not observed among the male doctor–patient combination. This means that an embarrassment of female patients could be stronger than male patients particularly in disclosing urinary symptoms to healthcare providers. The percentage of female urologists in Japan is only 8.2%, and it will be necessary to further promote the recruitment of female doctors in urology fields in order to encourage female patients with OAB to more actively visit doctors.

Overactive bladder (OAB) is associated with urinary incontinence and significantly reduces quality of life among the patients.^1^ Although anticholinergics and β3‐receptor stimulants are available as therapeutic agents, only about half of the patients with OAB respond adequately to oral medications alone, and satisfaction with the OAB treatment has been reported to be low.^2^ Previous studies have evaluated various factors associated with satisfaction with OAB treatment.^3^, ^4^ However, gender‐focused approach towards OAB treatment satisfaction has not been fully explored. In women, it may be more difficult than in men to appropriately communicate symptoms to their physicians due to embarrassment,^5^ and having a female doctor in charge of female patients may increase their treatment satisfaction. The primary goal of this study was to determine whether the gender combination of patient and physician may be associated with satisfaction with OAB treatment.

This questionnaire survey was conducted at Jyoban Hospital of Tokiwa Foundation in Iwaki, Fukushima, Japan. The hospital has one of the largest urology department in Fukushima prefecture with the daily outpatient visit of around 100 patients. We considered the adult patients aged 18 years or older who attended the outpatient office of the urology department of the hospital, were diagnosed with overactive bladder and had been taking anticholinergics or β3‐receptor stimulants, or both, for at least 3 months. Behavioural therapy is taught verbally or through pamphlets, as appropriate.

Our team developed the questionnaire referring to the previous studies covering this topic.^3^, ^4^, ^5^ The questionnaire was distributed to eligible patients between November 2020 and March 2021, and those unwilling to participants and those unable to complete the questionnaire on their own due to cognitive decline or visual impairment were excluded. In addition to the OAB treatment satisfaction, the questionnaire covered Overactive Bladder Symptom Scale (OABSS), International Prostate Symptom Score (IPSS), oral medications, effectiveness of OAB treatment, response to OAB symptoms and the medium and extent of information collection.

In the analysis, our primary outcome measure was OAB treatment satisfaction. In accordance with previous studies,^3^ we used the question of OAB treatment satisfaction to explore this outcome. This variable was transformed into a dichotomous measure (satisfied or other) for the following analyses. We coded the “other” response as the base outcome. First, univariate analyses were conducted using all variables as covariates and followed by multivariate analyses for significant factors. The main interest was the doctor–patient gender combination, which was categorized as male–male, female–female and other combinations and was forced into the final model. The study was approved by the hospital ethics committee and was conducted with the consent of the patients (Study No. 2020‐009).

A total of 147 patients participated in the study (Data S1). In summary, 91 (61.9%) were male, and the mean age was 73.5 years (standard deviation 9.9 years). The urologists who provided these outpatient services were two women and six men, of whom two women and three men were certified urologists. According to the multivariable logistic regression analysis of the OAB treatment satisfaction (Table 1), compared to when the gender of doctor and patient was not the same, female patients tended to be significantly more satisfied when they were treated by female doctors (odds ratio 10.79, 95% confidence interval 1.27–92.05). On the other hand, no similar trend was observed when male patients were treated by male doctors (odds ratio 1.26, 95% confidence interval 0.25–6.34).

**TABLE 1:** Multiple logistic regression model for satisfaction for overactive bladder management.

Variables	Odds ratio	*P*‐value	CI
Sex			
Male	1.00		
Female	1.27	0.799	(0.20–7.85)
Gender matching (doctor–patient)			
Male–male	1.26	0.783	(0.25–6.34)
Female–female	10.79	**0.030** *	(1.27–92.05)
Opposite	1.00		
Effectiveness			
Effective	14.45	**0.018** *	(1.59–131.20)
Neutral	0.15	0.213	(0.01–3.02)
Not effective	1.00		
OABSS	0.91	0.340	(0.76–1.10)
IPSS score			
Mild symptom	1.00		
Moderate symptom	0.32	0.119	(0.07–1.34)
Severe symptom	0.03	**0.012** *	(0.00‐0.46)
Information acquisition behaviour			
Never acquired	1.00		
Seeking behaviour	1.08	0.937	(0.16–7.08)
Scanning behaviour	0.97	0.968	(0.25–3.75)
Drug			
β_3_‐Adrenoceptor agonists only	1.00		
Anticholinergics only	1.71	0.525	(0.33–9.02)
Both			
Consultation	1.00		
No	0.25	0.528	(0.00–18.84)
Yes			
Counterplans	1.00		
No	0.38	0.165	(0.10–1.49)
Yes	0.31	0.100	(0.08–1.25)

*Note*: Five‐scale Likert scale was divided into 2 as 0 = *unsatisfied/neutral* and 1 = *satisfied*.

* Statistically significant (less than 0.05).

In the present study, which examined doctor–patient gender combinations in satisfaction with OAB treatment, as hypothesized, satisfaction was higher for female doctor–female patient combinations compared with different doctor–patient genders. This is an extremely important finding for the future of overactive bladder (OAB) treatment in Japan, as there have been no similar studies.

A notable fact was that similar associations were not observed among the male doctor–patient combination. This means that an embarrassment of female patients could be stronger than male patients particularly in disclosing urinary symptoms to healthcare providers. In Japan, it is estimated that female OAB patients account for 10.8% of those over 40 years of age, but less than half of them reportedly seek medical consultation.^6^ Further, the main reason for such avoidance is speculated to be embarrassment. The percentage of female urologists in Japan is only 8.2%, and it will be necessary to further promote the recruitment of female doctors in urology fields in order to encourage female patients with OAB to more actively visit doctors. Limitations of the study include a small sample size. In addition, the regression analysis may not have fully adjusted for the effects of confounding factors; for example, the regression analysis did not take into account whether or not behavioural therapy was used. However, this is due to the limited number of physicians available in an area where there is a severe shortage of physicians.

## AUTHOR CONTRIBUTIONS

All the authors contributed to the conception and designing of the article. Yukiko Kouchi wrote the manuscript. All the other authors did a critical review of the article. All the authors read the final manuscript and approved its submission.

## CONFLICT OF INTEREST STATEMENT

The authors declare that they have no conflict of interest.

Approval of the research protocol by an Institutional Reviewer Board and the approval number: 2020–009.

## ETHICS STATEMENT

All human subjects provided written informed consent with guarantees of confidentiality.

## Supporting information


**Data S1.** Patient characteristics (N = 147)
