# Supplementary material for: An examination of the relationship between satisfaction with overactive bladder (OAB) treatment and the doctor–patient gender: A questionnaire‐based single‐institution study
Source: BJUI Compass. 2023 Mar 25;4(4):417–9. doi: 10.1002/bco2.236 (PMC10268576; doi:10.1002/bco2.236)
Supplement: Supplementary file 1 — Data S1. Patient characteristics (N = 147) [file BCO2-4-417-s001.docx]

**Supplementary Material 1. Patient characteristics (N=147)**

| **Variables** | **n** | **%** |
| --- | --- | --- |
| **Gender** |  |  |
| Male | 91 | 61.9 |
| Female | 56 | 38.1 |
| **Age [mean (SD)]** | [73.5 (9.9)] | |
| < 60 | 12 | 8.2 |
| 60 to 69 | 28 | 19.0 |
| 70 to 79 | 69 | 46.9 |
| > 79 | 38 | 25.9 |
| **Perceived efficacy rate** |  |  |
| Very effective | 17 | 11.6 |
| Effective | 60 | 40.8 |
| Neutral | 44 | 29.9 |
| Ineffective | 22 | 15.0 |
| Very ineffective | 4 | 2.7 |
| **Satisfaction rate** |  |  |
| Very satisfied | 12 | 8.2 |
| Satisfied | 33 | 22.4 |
| Neutral | 53 | 36.1 |
| Dissatisfied | 40 | 27.2 |
| Very dissatisfied | 9 | 6.1 |
| **Advisors** |  |  |
| Parents | 5 | 3.4 |
| Children | 30 | 20.4 |
| Siblings | 18 | 12.2 |
| Friends | 20 | 13.6 |
| Medical staff | 105 | 71.4 |
| Others | 29 | 19.7 |
| None | 7 | 4.8 |
| **Counterplans for symptoms** | |  |
| Changing underwear | 40 | 27.2 |
| Use of pads/diapers | 63 | 42.9 |
| Use of tissue | 14 | 9.5 |
| Others | 8 | 5.4 |
| No counterplan | 33 | 22.4 |
| **Information acquisition** |  |  |
| Seeking behavior | 80 | 54.4 |
| Scanning behavior | 87 | 59.2 |
| Never acquired | 42 | 28.6 |
| **OABSS [mean (SD)]** | [6.38 (3.02)] | |
| **IPSS (n=146)** |  |  |
| Mild symptom | 26 | 17.7 |
| Moderate symptom | 93 | 63.3 |
| Severe symptom | 27 | 18.4 |
| **Medicine** |  |  |
| β_3_-adrenoceptor agonists only | 107 | 72.8 |
| Anticholinergics only | 16 | 10.9 |
| Both | 24 | 16.3 |
| **Doctor-patient gender matching** | |  |
| Male-Male | 70 | 47.6 |
| Female-female | 23 | 15.7 |
| Opposite | 54 | 36.7 |

SD: standard deviation; OABSS: overactive bladder symptoms score; IPSS: International prostate symptom score
